# Quantification of Gut Microbiota Dysbiosis-Related Organic Acids in Human Urine Using LC-MS/MS

**DOI:** 10.3390/molecules27175363

**Published:** 2022-08-23

**Authors:** Yu-Tsung Lee, Sui-Qing Huang, Ching-Hao Lin, Li-Heng Pao, Chun-Hui Chiu

**Affiliations:** 1 Graduate Institute of Health Industry and Technology, Research Center for Food and Cosmetic Safety, College of Human Ecology, Chang Gung University of Science and Technology, Taoyuan 33303, Taiwan; 2 Department of Gastroenterology and Hepatology, Linkuo Chang Gung Memorial Hospital, Taoyuan 33305, Taiwan; 3 Department of Traditional Chinese Medicine, Chang Gung Memorial Hospital, Keelung 20401, Taiwan

**Keywords:** gut microbiota, LC-MS/MS, organic acid, human urine

## Abstract

Urine organic acid contains water-soluble metabolites and/or metabolites—derived from sugars, amino acids, lipids, vitamins, and drugs—which can reveal a human’s physiological condition. These urine organic acids—hippuric acid, benzoic acid, phenylacetic acid, phenylpropionic acid, 4-hydroxybenzoic acid, 4-hydroxyphenyl acetic acid, 3-hydroxyphenylpropionic acid, 3,4-dihydroxyphenyl propionic acid, and 3-indoleacetic acid—were the eligible candidates for the dysbiosis of gut microbiota. The aim of this proposal was to develop and to validate a liquid chromatography–tandem mass spectrometry (LC-MS/MS) bioanalysis method for the nine organic acids in human urine. Stable-labeled isotope standard (creatinine-d_3_) and acetonitrile were added to the urine sample. The supernatant was diluted with deionized water and injected into LC-MS/MS. This method was validated with high selectivity for the urine sample, a low limit of quantification (10–40 ng/mL), good linearity (*r* > 0.995), high accuracy (85.8–109.7%), and high precision (1.4–13.3%). This method simultaneously analyzed creatinine in urine, which calibrates metabolic rate between different individuals. Validation has been completed for this method; as such, it could possibly be applied to the study of gut microbiota clinically.

## 1. Introduction

Urine analysis is an important indicator that is used to assess human health as it contains many metabolic breakdown products from a wide range of foods, drinks, drugs, environmental contaminants, endogenous waste metabolites, and bacterial byproducts [1], which disclose many physiological and health indicators. Metabolites produced by the intestinal flora after metabolizing food residues, such as organic acids, are absorbed through the intestinal tract, metabolized, and excreted in urine [2]. Previous studies have found that the content of organic acids detected in urine is related to diet [3] and chronic conditions, such as allergies [4], diabetes [5], and obesity [6].

Benzoic acid (BA) is a product of phenylalanine [7] and polyphenol metabolism [8] from intestinal bacteria. In addition, BA is also used as an additive for preservative applications in food [9]. After absorption by the intestine, BA is transferred to the liver and detoxified by combination with glycine to form hippuric acid (HA), which is then excreted from urine [8]. Elevated levels of benzoic acid in urine are associated with glycine deficiency or abnormal liver function [10]. Some studies have shown that exposure to toluene in occupational environments leads to an increase in urinary HA [11]. In patients with type 2 diabetes, decreased levels of HA are associated with obesity and hypertension [5,6]. Phenylacetic acid (PAA) and phenylpropionic acid (PPA) are products of phenylalanine metabolism caused by intestinal bacteria [7,12,13]. High levels of PAA or PPA in urine may result from the dysbiosis of intestinal flora [7] or the decreased metabolism of phenylalanine, such as phenylketonuria (PKU) [14]. PAA is also a metabolite of 2-phenylethylamine, and low levels of PAA in urine are considered as a marker of depression [15]. Urinary 4-hydroxybenzoic acid (4-HBA) and 4-hydroxyphenylacetic acid (4-HPAA) represent a considerable percentage of tyrosine intake [7]. 4-HBA is one of the major catechin metabolites after the intake of green tea infusions [16]. In addition, 4-HPAA has been found to be useful in screening for small bowel diseases associated with anaerobic bacterial overgrowth [17]. 3-hydroxyphenylpropionic acid (3-HPPA) is a major metabolite of ingested proanthocyanidins and chlorogenic acid caused by colonic bacteria [18,19]. High levels of 3-HPPA may indicate the increased intestinal bacterial metabolism of dietary polyphenols. 3,4-dihydroxyphenyl propionic acid (3,4-DHPPA) is produced from dietary quinolones, such as naringin, by various clostridial species, and elevated levels of 3,4-DHPPA may imply Clostridium overgrowth [2,19]. 3-Indoleacetic acid (IAA) is a breakdown product of tryptophan metabolism produced by the action of gut bacteria, such as *Bifidobacterium* and *Bacteroides* [12,20,21]. IAA frequently occurs at low levels in urine and has been found in elevated levels in urine of patients with phenylketonuria or diet change [22]. Creatinine is usually produced at a fairly constant rate by muscle tissues and is applied to compensate for the different rates of metabolism between individuals [1]. The kidney has the capacity to regulate amount of fluid within the body, hence the urinary concentration of any compound is dependent on the compound excretion rate and the urinary flow rate. The most common normalization method that urinary creatinine is used for is to adjust for quantitation of urine analyte. The range of concentrations of these organic acids and creatine in human urine are summarized in Figure S1.

Urinary organic acids are diverse, and their content varies in a wide range (approximately 0.5–250 μmol/mmol creatinine [1]). Previous studies using capillary electrophoresis [23], nuclear magnetic resonance (NMR) [24], capillary electrophoresis–mass spectrometry (CE-MS) [25], or gas chromatography–mass spectrometry (GC-MS) [26,27] for organic acid detection in urine have been performed. Sample preparation for NMR is relatively easy, but NMR shows poorer sensitivity and specificity than MS [28]. CE-MS can analyze highly polar and charged compounds, but it shows poor method robustness and stability [29,30,31]. GC-MS is a highly sensitive and selective analytical instrument and is extensively employed to identify and quantify urinary metabolites. However, sample preparation for GC-MS is time-consuming due to derivatization [7,27]. Liquid chromatography–tandem mass spectrometry (LC-MS/MS) is another popular instrument with high sensitivity and selectivity. In addition, sample preparation for LC-MS/MS is quick and easy compared to GC-MS; therefore, LC-MS/MS is widely employed to analyze metabolites in urine. Obrenovich et al. (2017) [32] used LC-MS/MS to quantify several compounds in urine samples, including IPA and 3,4-DHPPA, but not for the analysis of gut microbiota-related organic acids. However, in recent studies, LC-MS/MS was used to analyze several aromatic amino acid-derived microbial metabolites in rat serum, urine, and feces [33]. Most studies analyze a few organic acids in human samples by LC-MS/MS, but there has been no research conducted which involves analyzing intestinal flora-related urinary organic acids in human samples. The matrix effect is one of the most important issues in LC-MS analysis. Signal enhancement or suppression has often occurred in ESI due to the co-eluting endogenous components in the sample matrix, which may facilitate or compete with the ionization of analytes. The European Medicines Agency (EMA) recommends using a stable-labeled isotope standard (as an internal standard), which is an easy and effective method to overcome the matrix effect. In this study, we developed a simple, rapid, and highly sensitive method by LC-MS/MS to analyze nine intestinal flora-related urinary organic acids, namely, HA, BA, PAA, PPA, 4-HBA, 4-HPAA, 3-HPPA, 3,4-DHPPA, IAA, and creatinine. This developed method was also validated based on the biological method validation guidelines of the EMA [34,35].

## 2. Results and Discussion

### 2.1. LC-MS/MS Modifier Optimization

The organic eluent was optimized and studied as follows. Compared with acetonitrile and methanol, methanol showed lower background signals and better peak shapes of organic acids with LC-MS/MS. Hence, methanol was chosen as the mobile phase [33]. Table 1 shows the negative-ion electrospray ionization (ESI) responses of nine organic acids and positive-ion ESI of creatinine in the mobile phase with formic acid or acetic acid added as modifiers. Except for HA, 4-HBA, and 3,4-DHPPA, increases in responses were observed for all analytes when acetic acid was added to the mobile phase compared to formic acid. Overall, the negative-ion responses decreased gradually with an increasing acid concentration. The poor sensitivity of PAA was also improved by acetic acid, which enhanced the negative-ion ESI response by approximately 11.6 times. Although stronger ESI responses of HA, 4-HBA, and 3,4-DHPPA were observed when formic acid was added into the mobile phase, the responses were all enough to meet the sensitivity requirement, regardless of whether formic acid or acetic acid was used as a modifier. Acetic acid was selected as a more suitable modifier in the following applications for a better signal intensity of PAA.

**Figure 1:**
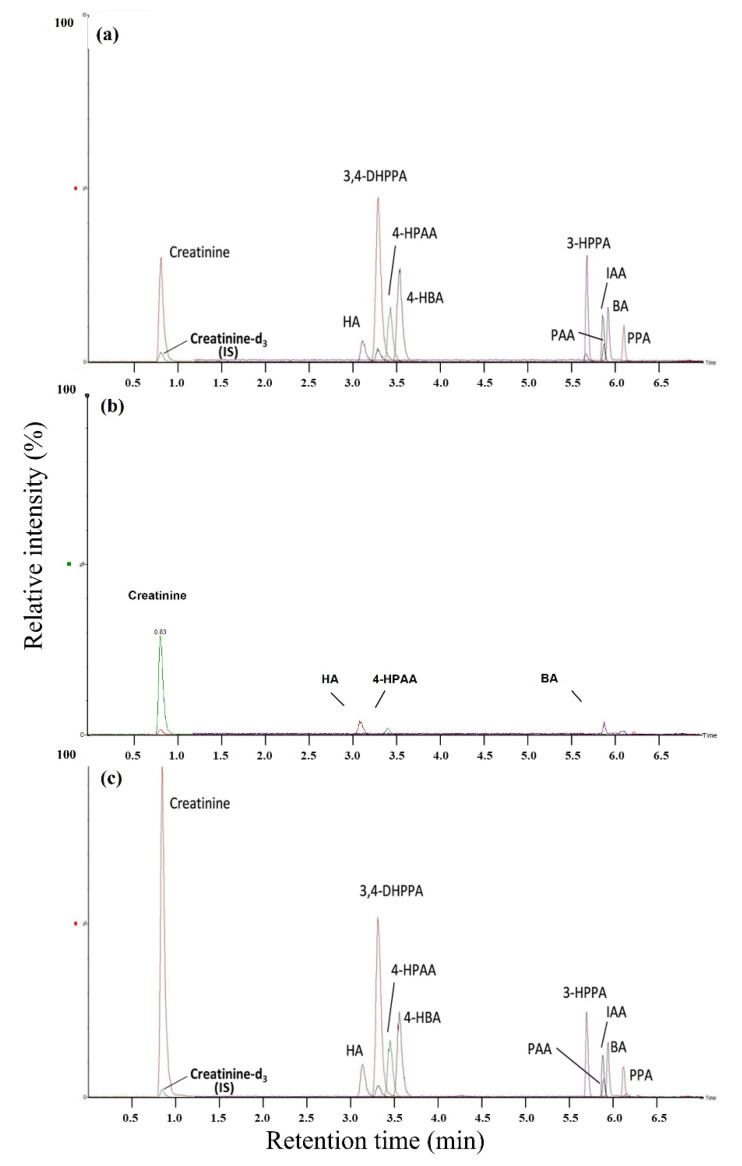
Mass chromatography of 10 analytes and internal standard (IS) in (**a**) 2% acetonitrile, (**b**) urine, and (**c**) a urinary extract. The spiked concentration of 10 analytes was equal to medium quality control (MQC), as shown in Table 2. Extracted ion chromatograms (EICs) of 11 multiple reaction monitoring (MRM) transitions were overlapped, and the highest EIC peak was defined as 100% (6.50 × 10^6^).

Negative-ion ESI response in reversed-phase chromatography was affected by the pKa of analytes and pH of the mobile phase. The pKa of nine organic acids ranged from 3.59 to 4.73 [36], which were all above the pH (2.95–3.45) of tested modifiers (Table 1). It could be inferred that the nine organic acids favored a non-ionized form in the acidic mobile phase below their pKa; however, only a few moieties existed in ionized form. This is consistent with the general idea that the non-ionized analytes contribute to better chromatographic performance in reversed-phase LC. On the other hand, intense [M-H]^−^ ion during the ESI of acidic eluents could be explained by wrong-way-round ionization. The ionization of these nine organic acids may take place via a gas-phase proton transfer reaction [37]. Acetate produced anions with higher gas-phase proton affinity than formate and showed a greater tendency to deprotonate acidic analytes [38]. Similar results were observed in that acetic acid solutions were more effective compared to formic acid solutions when attempting to enhance the negative-ion ESI responses of phenolic compounds [39]. Another interesting finding was that the response of PAA was much higher in PAA-only solution (1.48 × 10^8^) than in a ten-analyte mixed solution (9.53 × 10^6^). Figure 1 indicates that the peak of PAA overlapped with that of BA and IAA; that is, these three analytes would be subject to electrospray in a small-time region and would compete with one another to be ionized. The results suggested that the ionization efficiency of PAA was seriously suppressed by the co-occurrence of BA and IAA, and that this competition effect led to a response drop.

### 2.2. Method Validation

Figure 1 shows an overlapping MRM chromatogram for ten analytes and an internal standard (IS) in 2% acetonitrile and a urine sample. Each extracted ion chromatogram (EIC) showed that all analytes of interest were easily differentiated in a single MRM transition, and no interferences were observed from endogenous urine components or other analytes near their established retention times. These aromatic acids were hardly separated or time-consuming in an HPLC method for their similar structures and chemical properties. All analyte identification was achieved from the retention time, molecular ions, and fragmentation pattern for the quantitative determination. PAA, BA, and IAA had the same retention times at 5.9 min, which were detected separately based on their differential molecular ions at 122, 135, and 174 m/z, respectively, at different channels in the detector. Furthermore, their differential molecular ions and product ions were quantified using the MRM mode (Table 3); the same retention time did not affect the quantitative results (Figure S2). The MRM transitions of LC-MS/MS were highly selective for analytes with specific molecular weights, which efficiently saved analysis time in the chromatographic separation.

Signal enhancement or suppression often occurred in ESI due to the co-eluting endogenous components in the sample matrix, which may facilitate or compete with the ionization of analytes. Therefore, the evaluation of matrix effects was quite critical for the development of an ESI-MS-based analytical method. Since some of these compounds are endogenous compounds, there is no real blank sample for urine. According to the EMA guidelines [34,35], the standard addition method should be applied to spike samples to calculate the recovery and confirm the accuracy of this method. The coefficient of variation (CV) value of all MFs and IS-normalized MFs at low quality control (LQC) and high quality control (HQC) concentrations from six samples were below 15%, which met the EMA criterion and showed that the matrix effects between different samples were similar. The MFs of nine organic acids ranged from 91.9 to 107.8% (Table 4), which indicated that the matrix effects of these analytes were negligible. If all nine organic acids underwent the same sample preparation process, HA and 3,4-DHPPA had an obvious matrix effect (data not shown). Due to the high sensitivity of LC-MS/MS (Table S1) and the high urinary contents of HA and 3,4-DHPPA, a lower sampling volume (100 µL instead of 1 mL of urine) could effectively reduce the matrix effect and maintain the same detection limit. Low MF at LQC (27.7%) and HQC (22.9%) for creatinine suggested strong signal suppression in the urinary matrix. This matrix effect could be successfully corrected by creatinine-d_3_ as IS, and the IS-normalized MFs of creatinine were 108.3% at LQC and 100.8% at HQC.

This lower limit of quantification (LLOQ) (s/n >10) and linearity (*r* > 0.99) was proposed by EMA. The limit of detections (LOD) ranged from 0.5 to 2 ng/mL (data not shown). The linear ranges were based on analytical performance and fitted the guidelines. The S/N ratios of analytes at LLOQ were all greater than ten. Each calibration curve was analyzed, the *r* values of which were all higher than 0.99 (Table 5). Weighted 1/x linear regression was selected for quantification due to its better precision near lower concentrations. The accuracy of each level was estimated by the ratio of the back-calculated concentration to the nominal concentration, and all of them met the criterion of 85–115% for each level (80–120% for LLOQ). The results indicated good linearity and accuracy over the selected range. According to the AGREE-Analytical GREEnness Metric Approach score analysis method to evaluate the degree of green analytical chemistry [40], the value for our method is 0.48 (values close to 1 indicate that the analysis method is greener). In addition, this method simultaneously analyzed creatinine and nine gut microbiota dysbiosis-related organic acids in human urine. These results indicate that the creatinine calibrates metabolic rate between different individuals at the same time, with no need for extra creatine testing. The literature claims that the detection limit for 20 organic acids ranged between 1.08 to 32.4 ng/mL in rat urine, and that precision values were below 15% and accuracies ranged from 85% to 115% for all analytes [41]. None of the current methods can simultaneously determine all of the gut microbiota metabolites in human urine matrices. LC-MS/MS was applied for the quantification of related microbial metabolites; however, time-consuming solid-phase extraction was used for sample pretreatment [42], or there was no normalization of urinary creatinine concentrations [33]. A novel and validated LC/MS/MS method with simple and fast sample preparation produced for the determination of nine gut microbial-related organic acids in human urine was established in the present study.

In the carryover assay, no peak corresponding to creatinine, PAA, PPA, 4-HBA, 4-HPAA, or IAA was detected in the blank urinary extract, which was immediately injected after a ULOQ sample. However, weak responses for HA, BA, 3-HPPA, and 3,4-DHPPA were observed in 0.52%, 11.98%, 1.12%, and 0.75% in the LLOQ sample, respectively. The results indicated that the carryover of all analytes was quite minor (<2%), except for BA, and all of them met the criterion (<20%).

Within-run and between-run accuracy and precision were calculated for the LLOQ, LQC, medium quality control (MQC), and HQC concentrations (Table 2). The calculated within-run and between-run accuracy values ranged from 85.8 to 110.3%, with the majority being >90%. The lowest accuracy (85%) for PAA was likely due to the poor sensitivity. All calculated within-run and between-run precision values were <15%. These results all met the EMA criterion and demonstrated the high accuracy and repeatability of this analytical method.

In the dilution integrity assay, analytes were spiked in a blank sample to obtain the concentration equivalent of five times that of ULOQ. Before injection, the prepared spiked urinary extracts were diluted ten times with blank extract. The precision and accuracy of the dilution integrity ranged from 1.5 to 3.6% and from 97.3 to 105.2% for all analytes, which all passed the EMA criterion of <15% and 85–115%, respectively. The results indicated that a sample concentration above the ULOQ could be successfully diluted.

**Figure 2:**
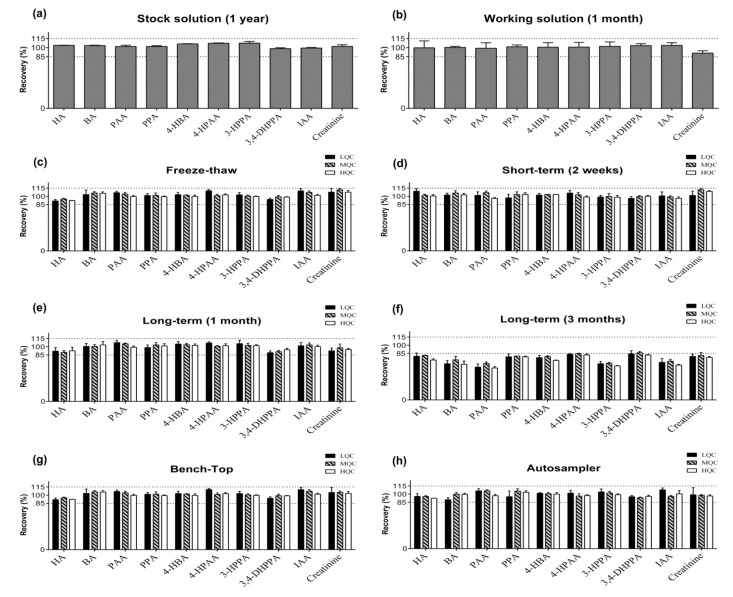
The stability studies of nine organic acids and creatinine in urine or working solution, including (**a**) stock solution stability after one year, (**b**) working solution stability after one month, (**c**) freeze–thaw stability after three cycles, (**d**) short-term stability after two weeks, long-term stability after (**e**) one month and (**f**) three months, (**g**) bench-top stability after 6 h, and (**h**) autosampler stability after 8 h. All stability studies were performed with three determinations for each (*n* = 3).

The stability of the analytes in urine under different temperatures and timing conditions, as well as in stock solution and working solution, is shown in Figure 2a,b. The stability of stock solutions was stable for all analytes at −30 °C for one year, and the recovery ranged from 98.4 to 107.4%. The stability of working solutions was also stable for one-month storage, and the recovery were ranged from 91.0 to 103.8%. These results indicated that the prepared solutions were stable throughout one year and one month for the stock solution and working solution, respectively, which all fulfilled the EMA acceptable criterion that the mean concentration at each level should be within ±15% of the nominal concentration [34,35].

For freeze–thaw stability determination, QC samples were subjected to three freeze and thaw cycles. The recoveries of all analytes at three concentrations ranged from 91.6 to 111.9%, which all passed the EMA criterion of 85–115% (Figure 2c). The results suggested that all analytes were stable, regardless of temperature variation.

For short- and long-term stability determination, the stored samples were tested after storage in a freezer for two weeks, one month, and three months. The recoveries of all analytes for two weeks and one month were all within the EMA criterion of 85–115%. However, the recoveries of almost all analytes after three-month storage in a freezer decreased to <85% (Figure 2d–f). The findings revealed that samples stored in a freezer (−30 °C) should be analyzed within one month after sampling to ensure accuracy.

The stability of QC samples left on the bench for 6 h and in the autosampler for 8 h was assessed to simulate the conditions encountered during routine sample preparation, respectively. The data representing the bench-top and autosampler stability are shown in Figure 2g,h, which were all within the EMA acceptance criterion of ±15%. In this study, only creatinine-d3 was used as the IS for all analytes. Although creatinine/creatinine-d3 is analyzed in positive mode and other compounds are analyzed in negative mode, the results of calibration curve linearity and validation revealed the applicability of the setting. These results showed reliable stability under the regular analytical procedure.

## 3. Material and Methods

### 3.1. Chemicals and Reagents

HA, PAA, PPA, 4-HBA, IAA, 3,4-DHPPA, creatinine, and creatinine-d_3_ were purchased from Sigma-Aldrich (Saint Louis, MO, USA). BA and 4-HBA were purchased from Tokyo chemical industry (Tokyo, Japan). 3-HPPA was purchased from Alfa Aesar (Ward Hil, MA, USA). Formic acid, acetic acid, acetonitrile, and methanol (LCMS grade) were obtained from JT Baker (Warren, PA, USA). Deionized water (18.2 MΩ·cm at 25 °C, TOC ≤ 10 ppb) for LC-MS/MS was produced by the Millipore Direct-Q^®^ Water Purification System (Millipore, Billerica, MA, USA).

Separate stock solutions of ten analytes and creatinine-d_3_ (10,000 µg/mL) were prepared in 50% methanol and stored at −30 °C until use. A stock solution of creatinine-d_3_ was further diluted to 100 µg/mL as an IS solution for sample preparation. A working solution at concentrations of 100 (BA, 4-HBA, PPA, IAA, and 3-HPPA), 200 (4-HPAA), 400 (HA, PAA, 3,4-DHPPA), and 1000 µg/mL (creatinine) was diluted from stock solutions in 50% methanol.

### 3.2. Sample Preparation

Urinary specimens (800–1400 mL) from six volunteers were collected separately within 24 h and used in the following studies. These volunteers were healthy graduated students who did not take any medicine.

The HA and creatinine were analyzed apart from the other eight analytes due to having very high concentrations as compared to other analytes in urine. The content of organic acids in urine and the content of creatinine are widely distributed; in particular, the content of creatinine and HA is extremely high. Therefore, HA and creatinine must be diluted with high multiples during sample preparation. The sample preparation was divided into two parts (low dilution using 1 mL human urine and high dilution using 0.1 mL human urine), but the same analytical conditions were used for the quantitative analysis. For BA, PAA, PPA, 4-HBA, 4-HPAA, 3-HPPA, IAA, and 3,4-DHPPA determination, an aliquot of 10 µL IS solution (100 µg/mL creatinine-d_3_) was added to 1 mL human urine in a tube (low dilution). For HA and creatinine determination, an aliquot of 10 µL IS was added to 0.1 mL human urine and 900 µL deionized water in a tube (high dilution). After vortex-mixing for 20 s, the mixture was added to 2 mL of acetonitrile and then the volume was made up to 10 mL with distilled water. The urine sample was shaken for 1 min and centrifuged (2100× *g* at 15 °C, 3 min). Then, 100 µL of supernatant was diluted with 900 µL of deionized water as an urinary extract. After filtration (nylon 0.22 μm), an aliquot of 5 μL filtrate was injected into the LC-MS/MS system. The detailed sample preparation procedure scheme is shown as Supplementary Materials in Figure S3.

### 3.3. Calibration Curves

The standard addition method was used to build the calibration curve. The calibrators for organic acid determination were prepared in urinary extracts by the addition of 10–200 µL working solution to the urine sample before sample preparation. Finally, eight-level calibration curves were in the range of 10–200 ng/mL for BA, 4-HBA, PPA, 3-HPPA, and IAA; 20–400 ng/mL for 4-HPAA; 40–800 ng/mL for HA, PAA, and 3,4-DHPPA; and 100–2000 ng/mL for creatinine. The detailed preparation method of the calibration curves is shown in Table S1.

### 3.4. Instrumental and Analytic Conditions

The LC-MS/MS system consisted of an ACQUITY APC™ UPLC system (Waters, Milford, CT, USA) and a triple quadrupole mass spectrometer Xevo TQ-S (Waters, Milford, CT, USA) equipped with an ESI source. MassLynx 4.1 software was used for instrument control and data analysis. The chromatographic method was developed using a Luna Omega C18 column (100 mm × 2.1 mm; 1.6 μm particle size; Phenomenex, CA, USA), fitted with a Security Guard Ultra holder for a UHPLC column (Phenomenex, CA, USA) at a column temperature of 35 °C. Mobile phases were performed using (A) aqueous solution and (B) methanol solution. Different volatile acids were used as follows: 0.025% formic acid (pH = 2.95) and 0.025% (pH = 3.45), 0.05% (pH = 3.29), and 0.1% acetic acid (pH = 3.14) were added, respectively, to both aqueous and methanol mobile phases to evaluate their effects on the MS/MS signal intensity of all analytes at a concentration of 1 µg/mL. The gradient program at a flow rate of 250 μL/min was as follows: 0–4 min, 25–25% B; 4–5 min, 25–90% B; 5–5.3 min, 90–99% B; 5.3–5.6 min, 99–99% B; 5.6–5.8 min, 99–25% B; and 5.8–7 min, 25–25% B. The mass parameters were modified according to Chiu et al., 2021 [43]. The ESI operated in positive mode for 0–1.2 min with 2.5 kV spray voltage, and in negative mode for 1.2–7 min with −1.5 kV. The parameter settings for ESI were as follows: desolvation gas, 400 L/h; cone gas, 20 L/h; nebulizer gas, 7.0 bar; desolvation temperature, 200 °C. All MS/MS data for analytes were collected in positive or negative ion modes via multiple reaction monitoring (MRM). The optimized MRM conditions are shown in Table 3. The urine concentrations of nine organic acids were normalized with urinary creatinine concentrations and are presented as µmol/mmol creatinine.

### 3.5. Method Validation

This bioanalytical method was validated to meet the criteria of selectivity, matrix effect, linearity, carryover, accuracy, precision, dilution integrity, and stability proposed by the EMA [34,35]. In the following studies, four QC samples were prepared using blank urine samples spiked at different concentrations, namely, LLOQ, LQC, MQC, and QC HQC. The concentrations of LQC, MQC, and HQC for all analytes were equal to 3, 9, and 16 times of the LLQC, respectively.

The selectivity of the method was investigated by comparing chromatograms of urinary extract and spiked urinary extract with ten analytes and IS to ensure that all analytes of interest could be easily differentiated and were free of interference.

The matrix effect of LC-MS/MS for each analyte was evaluated by the matrix factor (MF) or IS-normalized MF. In brief, two sets of solutions were prepared. Set A consisted of analytes and IS in the 2% acetonitrile; set B consisted of urinary extract spiked with analytes and IS after extraction. Each set was prepared at two concentrations, namely, LQC and HQC, in six replicates from six people. The peak area of analytes and IS was used to calculated MF and IS-normalized MF according to the following formula:

MF (%) = B/A × 100%

IS-normalized MF = MF_analytes_/MF_IS_ × 100%

where A and B are the peak area of each analyte and IS from set A and B, respectively. The mean and CV of MFs and IS-normalized MFs were calculated to evaluate the matrix effect and its variation for each analyte (*n* = 6).

Linearity was evaluated for each analyte over the concentration range specified in Table 5. Each calibration curve was generated by a weighted linear regression data fit (w = 1/x) in which the peak area (nine organic acids) or the peak area ratios (creatinine) of the calibrators were plotted against their concentrations. The peak area ratio was the area of creatinine divided by that of the creatinine-d_3_. LLOQ was determined as the lowest concentration level of the calibration curve, and the signal-to-noise (S/N) ratio of all analytes at the LLOQ concentration had to be greater than ten. The back-calculated concentrations of each calibrator had to be within ±15% (within ± 20% for LLOQ) of the nominal value, and the correlation coefficient (*r*) of linear calibration curves was calculated to evaluate the linearity. Carryover was assessed by injecting a blank urine sample after a ULOQ sample. Carryover was considered acceptable if the peak areas of all analytes and IS were less than 20% of those of the corresponding areas in the LLOQ sample.

Accuracy and precision were obtained by analyzing QC samples in within- and between-run assays. The within-run assay was determined within an analytical batch by analyzing six sample replicates at concentrations of LLOQ, LQC, MQC, and HQC for all analytes (Table 2). The within-run accuracy and precision were defined as the ratio of the calculated mean concentration to their corresponding nominal value and the CV from the six replicated QC samples (*n* = 6). The between-run assay was determined in three separate analytical batches on different days. The between-run accuracy and precision for a certain QC concentration were calculated in the same way as the within-run assay, but for a total of nine replicated QC samples (*n* = 9). The accuracy had to be within ±15% (within ±20% for LLOQ), and CVs could not exceed 15% (20% for LLOQ).

For the dilution integrity assay, urine samples were spiked with a high concentration of all analytes (1 µg/mL for BA, 4-HBA, PPA, 3-HPPA, and IAA; 2 µg/mL for 4-HPAA; 4 µg/mL for PAA, HA, and 3,4-DHPPA; and 10 µg/mL for creatinine). Each QC sample was diluted by blank extract before injection into LC-MS/MS. The calculated concentrations of diluted QC samples (*n* = 5) were used to calculate accuracy and precision as mentioned above.

The stability of all analytes in stock solutions and working solutions were assessed (*n* = 3) after one year and one month of storage at −30 °C, respectively. Freeze–thaw stability was assessed for fortified samples, which were stored over three freeze–thaw cycles (−30 °C for 12 h, and then room temperature for 1 h). Short- and long-term stability was assessed for fortified samples, which were stored for two weeks (short-term), one month, and three months (long-term) at −30 °C. Bench-top stability was assessed for fortified samples, which were left on the bench at room temperature for 6 h before sample preparation. All the fortified samples above were prepared by blank urine samples fortified at concentrations of LQC, MQC, and HQC (*n* = 3). Autosampler stability was assessed for blank urinary extracts fortified at LQC, MQC, and HQC concentrations (*n* = 3), which were left in the autosampler at 15 °C for 8 h before injection. All solutions for stability tests were analyzed after the specific storage conditions against freshly prepared calibration standards, and the obtained concentrations were compared to the nominal concentrations to calculate the recovery (%).

## 4. Conclusions

An LC-MS/MS method for the determination of nine gut microbiota-related organic acids and creatinine from fortified human urine was successfully developed and validated based on EMA regulatory guidelines. This method can analyze nine organic acids and creatinine in urine. Since the content of organic acids and creatinine in urine has a wide range, the main limitation of this study is that one sample was subjected to the same sample treatment twice (low dilution and high dilution). Although the calibration curve is linear and validated, the results show the suitability of this setup. Creatinine is a product of muscle tissue decomposition, excreted through the kidney filtration system. The concentration of creatinine in urine is a good indicator for the correction of the concentrations of metabolites among different individuals. The reliable, quick, and easy-to-perform method was found to be suitable for routine analysis in the clinical laboratory, which provided an approach for the further investigation of the diet, gut microbiota, and urinary organic acids.

## Figures and Tables

**Table 1:** Effects of formic acid and acetic acid on negative-ion ESI responses of nine organic acids and positive-ion ESI responses of creatinine at 1 µg/mL.

Analyte	Responses			
	0.025% FA (pH = 2.95)	0.025% AA (pH = 3.45)	0.05% AA (pH = 3.29)	0.1% AA (pH = 3.14)
HA	1.27 × 10^7^	2.01 × 10^6^	1.69 × 10^6^	1.64 × 10^6^
BA	3.92 × 10^5^	1.85 × 10^7^	1.5 × 10^7^	1.09 × 10^7^
PAA	8.21 × 10^4^	9.53 × 10^5^	9.11 × 10^5^	6.94 × 10^5^
PPA	5.26 × 10^5^	1.6 × 10^7^	1.24 × 10^7^	8.77 × 10^6^
4-HBA	2.47 × 10^7^	3.61 × 10^6^	2.92 × 10^6^	3.18 × 10^6^
4-HPAA	2.67 × 10^6^	7.87 × 10^6^	9.15 × 10^6^	6.18 × 10^6^
3-HPPA	1.46 × 10^7^	3.69 × 10^7^	3.25 × 10^7^	2.84 × 10^7^
3,4-DHPPA	1.77 × 10^7^	3.01 × 10^6^	8.57 × 10^6^	1.42 × 10^7^
IAA	9.65 × 10^6^	1.02 × 10^7^	1.46 × 10^7^	1.47 × 10^7^
Creatinine	1.69 × 10^6^	6.8 × 10^6^	6.21 × 10^6^	5.98 × 10^6^

Abbreviations are as follows: FA—formic acid; AA—acetic acid; HA—hippuric acid; BA—benzoic acid; PAA—phenylacetic acid; PPA—phenylpropionic acid; 4-HBA—4-hydroxybenzoic acid; 4-HPAA—4-hydroxyphenyl acetic acid; 3-HPPA—3-hydroxyphenylpropionic acid; 3,4-DHPPA—3,4-dihydroxyphenyl propionic acid; IAA—3-indoleacetic acid.

**Table 2:** Within-run and between-run accuracy and precision of nine organic acids and creatinine in human urine.

Analyte	Nominal Concentration (ng/mL)		Within-Run (*n* = 6)			Between-Run (*n* = 9)		
			Concentration (ng/mL)	Accuracy (%)	Precision (CV, %)	Concentration (ng/mL)	Accuracy (%)	Precision (CV, %)
HA	LLOQ	40	40.9 ± 5.5	102.3	13.3	38.5 ± 4.8	96.2	12.4
	LQC	120	126.9 ± 9.7	105.8	7.7	118.8 ± 11.7	99.0	9.8
	MQC	360	381.4 ± 18.9	105.9	5.0	377.4 ± 14.4	104.8	3.8
	HQC	640	662.5 ± 38.7	103.5	5.8	667.7 ± 29.0	104.3	4.3
BA	LLOQ	10	9.5 ± 0.9	94.6	9.9	9.4 ± 0.7	93.6	7.5
	LQC	30	29.5 ± 1.4	98.2	4.6	28.1 ± 1.7	93.6	6.2
	MQC	90	88.9 ± 2.8	98.8	3.1	88.5 ± 2.3	98.4	2.6
	HQC	160	159.7 ± 4.4	99.8	2.7	161.4 ± 4.7	100.9	2.9
PAA	LLOQ	40	34.4 ± 3.8	85.9	11.0	34.3 ± 3.0	85.8	8.7
	LQC	120	122.7 ± 6.9	102.3	5.7	124.4 ± 7.0	103.6	5.7
	MQC	360	372.3 ± 13.9	103.4	3.7	392.6 ± 26.9	109.1	6.8
	HQC	640	625.3 ± 22.2	97.7	3.6	634.0 ± 22.2	99.1	3.5
PPA	LLOQ	10	10.1 ± 0.7	100.6	6.5	9.7 ± 0.8	97.0	7.8
	LQC	30	29.7 ± 1.0	98.9	3.5	28.4 ± 1.8	94.6	6.3
	MQC	90	90.1 ± 3.2	100.1	3.5	88.2 ± 3.3	98.0	3.7
	HQC	160	161.6 ± 2.4	101.0	1.5	156.6 ± 6.1	97.9	3.9
4-HBA	LLOQ	10	9.7 ± 0.2	97.1	2.4	9.1 ± 0.7	91.0	7.9
	LQC	30	28.8 ± 1.0	96.1	3.5	28.0 ± 1.3	93.2	4.7
	MQC	90	89.6 ± 1.2	99.6	1.4	88.0 ± 2.3	97.8	2.6
	HQC	160	158.1 ± 2.7	98.8	1.7	157.1 ± 4.1	98.2	2.6
4-HPAA	LLOQ	20	18.6 ± 1.2	92.8	6.7	18.6 ± 1.4	93.0	7.4
	LQC	60	56.7 ± 2.3	94.5	4.1	55.0 ± 3.2	91.6	5.8
	MQC	180	179.3 ± 10.7	99.6	5.9	176.9 ± 8.3	98.3	4.7
	HQC	320	312.6 ± 18.4	97.7	5.9	317.7 ± 16.3	99.3	5.1
3-HPPA	LLOQ	10	9.7 ± 0.7	96.6	7.3	9.3 ± 0.7	92.9	7.8
	LQC	30	29.1 ± 0.8	97.0	2.9	29.0 ± 0.6	96.7	2.2
	MQC	90	89.8 ± 2.0	99.8	2.2	89.8 ± 1.9	99.8	2.2
	HQC	160	158.5 ± 4.4	99.1	2.8	158.7 ± 5.3	99.2	3.3
3,4-DHPPA	LLOQ	40	39.0 ± 3.4	97.6	8.7	36.3 ± 3.7	90.8	10.3
	LQC	120	111.9 ± 4.1	93.2	3.7	114.4 ± 3.9	95.3	3.4
	MQC	360	353.7 ± 10.7	98.3	3.0	358.8 ± 9.6	99.7	2.7
	HQC	640	622.9 ± 11.1	97.3	1.8	629.2 ± 16.8	98.3	2.7
IAA	LLOQ	10	9.9 ± 1.0	99.3	9.6	9.4 ± 0.9	93.8	10.1
	LQC	30	30.1 ± 2.2	100.3	7.2	29.4 ± 1.9	98.2	6.3
	MQC	90	90.3 ± 4.2	100.4	4.6	90.0 ± 3.2	100.0	3.6
	HQC	160	159.6 ± 3.5	99.7	2.2	159.0 ± 4.0	99.4	2.5
Creatinine	LLOQ	100	109.7 ± 9.8	109.7	9.8	109.3 ± 11.1	109.3	11.1
	LQC	300	324.9 ± 21.9	108.3	7.3	324.6 ± 30.3	108.2	10.1
	MQC	900	893.7 ± 43.2	99.3	4.8	992.7 ± 27.9	110.3	3.1
	HQC	1600	1613 ± 78.4	100.8	4.9	1754 ± 41.6	109.6	2.6

Abbreviations are as follows: CV—coefficient of variation; LLOQ—lower limit of quantification; LQC—low quality control; MQC—medium quality control; HQC—high quality control.

**Table 3:** MRM conditions for nine organic acids and creatinine.

Analyte	ESI Mode	Retention Time (min)	Q1 > Q3 (m/z)	Cone Voltage (V)	Collision Energy (eV)
HA	−	3.1	178 > 134	10	12
BA	−	5.9	121 > 77	44	12
PAA	−	5.9	135 > 91	10	10
PPA	−	6.1	149 > 105	36	12
4-HBA	−	3.6	137 > 93	10	14
4-HPAA	−	3.5	151 > 107	14	12
3-HPPA	−	5.7	165 > 121	10	12
3,4-DHPPA	−	3.3	181 > 137	10	12
IAA	−	5.9	174 > 130	10	14
Creatinine	+	0.9	114 > 44	34	25
Creatinine-d_3_ (IS)	+	0.9	117 > 47	44	25

**Table 4:** Matrix effects of nine organic acids and creatinine from six people (*n* = 6).

Analyte	Nominal Concentration (ng/mL)		Matrix Factor, MF (%)	CV (%)
HA	LQC	120	105.8	7.7
	HQC	640	103.5	5.8
BA	LQC	30	99.6	5.7
	HQC	160	98.9	4.6
PAA	LQC	120	94.3	6.2
	HQC	640	93.7	6.1
PPA	LQC	30	107.8	4.7
	HQC	160	105.3	3.7
4-HBA	LQC	30	105.8	4.3
	HQC	160	103.3	4.0
4-HPAA	LQC	60	97.5	9.2
	HQC	320	91.9	8.7
3-HPPA	LQC	30	105.8	4.7
	HQC	160	104.0	5.0
3,4-DHPPA	LQC	120	93.2	3.7
	HQC	640	97.3	1.8
IAA	LQC	30	105.3	7.9
	HQC	160	107.5	8.9
Creatinine	LQC	300	27.7	8.1
	HQC	1600	22.9	9.7
Creatinine	LQC	300	108.3 ^a^	7.8
	HQC	1600	100.8 ^a^	4.9

^a^ IS-normalized MF.

**Table 5:** Calibration curves and linearity assay of nine organic acids and creatinine.

Analyte	Calibration ^a^				Back-Calculated Concentration/Nominal Concentration ^b^ (%)							
	Range (ng/mL)	Slope	Intercept	*r*	1st Level	2nd Level	3rd Level	4th Level	5th Level	6th Level	7th Level	8th Level
HA	40–800	0.06 ± 0.01	−0.72 ± 0.47	0.9973–0.9994	108.00 ± 0.04	101.46 ± 0.03	98.52 ± 0.07	96.24 ± 0.04	94.89 ± 0.01	95.63 ± 0.03	100.47 ± 0.03	104.75 ± 0.02
BA	10–200	0.33 ± 0.06	0.56 ± 1.01	0.9979–0.9994	106.33 ± 0.03	98.00 ± 0.03	103.00 ± 0.04	94.39 ± 0.02	96.08 ± 0.04	99.00 ± 0.05	100.42 ± 0.03	102.72 ± 0.01
PAA	40–800	0.06 ± 0.02	−1.24 ± 0.38	0.9969–0.9979	114.33 ± 0.02	97.33 ± 0.06	96.54 ± 0.08	95.00 ± 0.05	94.02 ± 0.03	96.34 ± 0.04	101.69 ± 0.02	104.69 ± 0.01
PPA	10–200	0.21 ± 0.08	−0.57 ± 0.54	0.9956–0.9996	111.33 ± 0.07	95.83 ± 0.01	99.92 ± 0.02	95.56 ± 0.03	95.42 ± 0.03	96.97 ± 0.05	101.84 ± 0.05	103.13 ± 0.01
4-HBA	10–200	0.85 ± 0.37	−2.16 ± 1.22	0.9983–0.9993	108.00 ± 0.02	98.00 ± 0.02	99.92 ± 0.03	96.06 ± 0.02	96.08 ± 0.04	98.30 ± 0.03	100.67 ± 0.03	102.88 ± 0.00
4-HPAA	20–400	0.22 ± 0.07	−1.05 ± 0.72	0.9974–0.9991	109.17 ± 0.02	98.42 ± 0.02	100.46 ± 0.04	95.47 ± 0.03	94.52 ± 0.02	97.70 ± 0.04	100.61 ± 0.03	103.86 ± 0.00
3-HPPA	10–200	0.54 ± 0.14	−1.53 ± 0.59	0.9981–0.9995	108.33 ± 0.03	97.17 ± 0.02	98.67 ± 0.03	98.11 ± 0.04	96.67 ± 0.01	97.60 ± 0.03	100.47 ± 0.04	102.90 ± 0.03
3,4-DHPPA	40–800	0.29 ± 0.26	2.54 ± 2.79	0.9975–0.9982	97.75 ± 0.15	98.25 ± 0.07	102.83 ± 0.08	99.93 ± 0.06	104.65 ± 0.08	97.94 ± 0.05	98.89 ± 0.03	99.74 ± 0.04
IAA	10–200	0.15 ± 0.09	−0.09 ± 0.08	0.9992–0.9999	101.33 ± 0.05	98.33 ± 0.06	102.08 ± 0.01	96.72 ± 0.03	101.00 ± 0.02	101.70 ± 0.02	98.02 ± 0.02	100.88 ± 0.02
Creatinine	100–2000	0.26 ± 0.02	0.72 ± 2.15	0.9996–0.9998	98.50 ± 0.05	98.47 ± 0.03	101.59 ± 0.02	102.22 ± 0.02	101.20 ± 0.02	98.37 ± 0.02	100.34 ± 0.01	99.33 ± 0.01

^a^ Three calibration curves (*n* = 3) were used for calculation of slope, intercept, *r*, and accuracy of each level. ^b^ The nominal concentrations of level 1–8 were 10, 20, 40, 60, 80, 100, 150, and 200 ng/mL for BA, PPA, 4-HBA, 3-HPPA, and IAA; 20, 40, 80, 120, 160, 200, 300, and 400 ng/mL for 4-HPAA; 40, 80, 160, 240, 320, 400, 600, and 800 ng/mL for HA, PAA, and 3,4-DHPPA; and 100, 200, 400, 600, 800, 1000, 1500, and 2000 ng/mL for creatinine, respectively.

## Data Availability

Not applicable.

## Supplementary Materials

The following supporting information can be downloaded at: https://www.mdpi.com/article/10.3390/molecules27175363/s1, Figure S1: The range of concentrations of these organic acids and creatine in human urine; Figure S2: Representative MRM chromatograms of the nine organic acids, creatinine and creatinine-d3 (IS) standards; Figure S3: Sample preparation procedure scheme by gut microbiota dysbiosis related organic acids in urine; Table S1: The detailed preparation method of calibration curve [44,45].

## Abbreviations

3-HPPA—3-hydroxyphenylpropionic acid; 3,4-DHPPA—3,4-dihydroxyphenyl pro-pionic acid; 4-HBA—4-hydroxybenzoic acid; 4-HPAA—4-hydroxyphenylacetic acid; AA—acetic acid; BA—benzoic acid; CE–MS—capillary electrophoresis–mass spectrometry; CV—coefficient of varia-tion; ESI—electrospray ionization; EMA—European Medicines Agency; FA—formic acid; GC–MS—gas chromatography–mass spectrometry; HA—hippuric acid; HQC—high quality control; IS—internal standard; IAA—3-indoleacetic acid; LC-MS/MS—liquid chromatography–tandem mass spectrometry; LLOQ—lower limit of quantification; LOD—limit of detection; LQC—low quality control; MRM—multiple reaction monitoring; MQC—medium quality control; NMR—nuclear magnetic resonance; PAA—phenylacetic acid; PKU—phenylketonuria; PPA—phenylpropionic acid.
