# Supplementary material for: Quantification of Gut Microbiota Dysbiosis-Related Organic Acids in Human Urine Using LC-MS/MS
Source: Molecules. 2022 Aug 23;27(17):5363. doi: 10.3390/molecules27175363 (PMC9457824; doi:10.3390/molecules27175363)
Supplement: Supplementary file 1 [file molecules-27-05363-s001.zip › molecules-1825247-supplementary.pdf]

Title: Quantification of Gut Microbiota Dysbiosis-Related Organic Acids in Human Urine Using LC-MS/MS

Authors and affiliations:

Yu-Tsung Lee[1], Sui-Qing Huang[1], Ching-Hao Lin[1], Li-Heng Pao[1,2*] and Chun-Hui Chiu[1,3*]

[1] Graduate Institute of Health Industry and Technology, Research Center for Food and Cosmetic Safety, College of Human Ecology, Chang Gung University of Science and Technology, Taoyuan City 33303, Taiwan

[2] Department of Gastroenterology and Hepatology, Linkuo Chang Gung Memorial Hospital, Taoyuan City 33305, Taiwan

[3] Department of Traditional Chinese Medicine, Chang Gung Memorial Hospital, Keelung City 20401, Taiwan

* Correspondence

Supplementary information

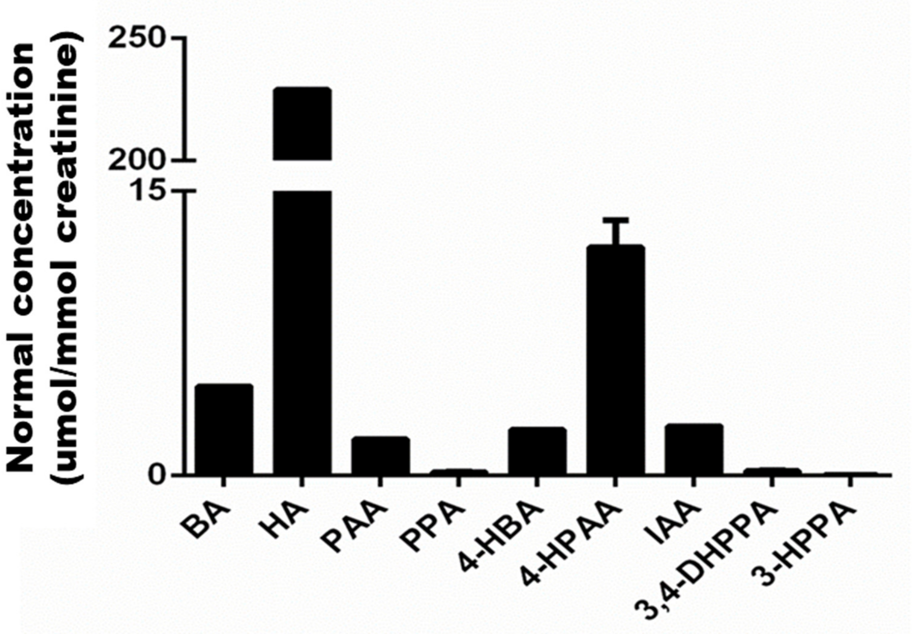

Figure S1. The range of concentrations of these organic acids and creatine in human urine.[1-3]

Abbreviations were as following: HA for hippuric acid, BA for benzoic acid, PAA for phenylacetic acid, PPA for phenylpropionic acid, 4-HBA for 4-hydroxybenzoic acid, 4-HPAA for 4-hydroxyphenyl acetic acid, 3-HPPA for 3-hydroxyphenylpropionic acid, 3,4-DHPPA for 3,4-dihydroxyphenyl propionic acid, and IAA for 3-indoleacetic acid.

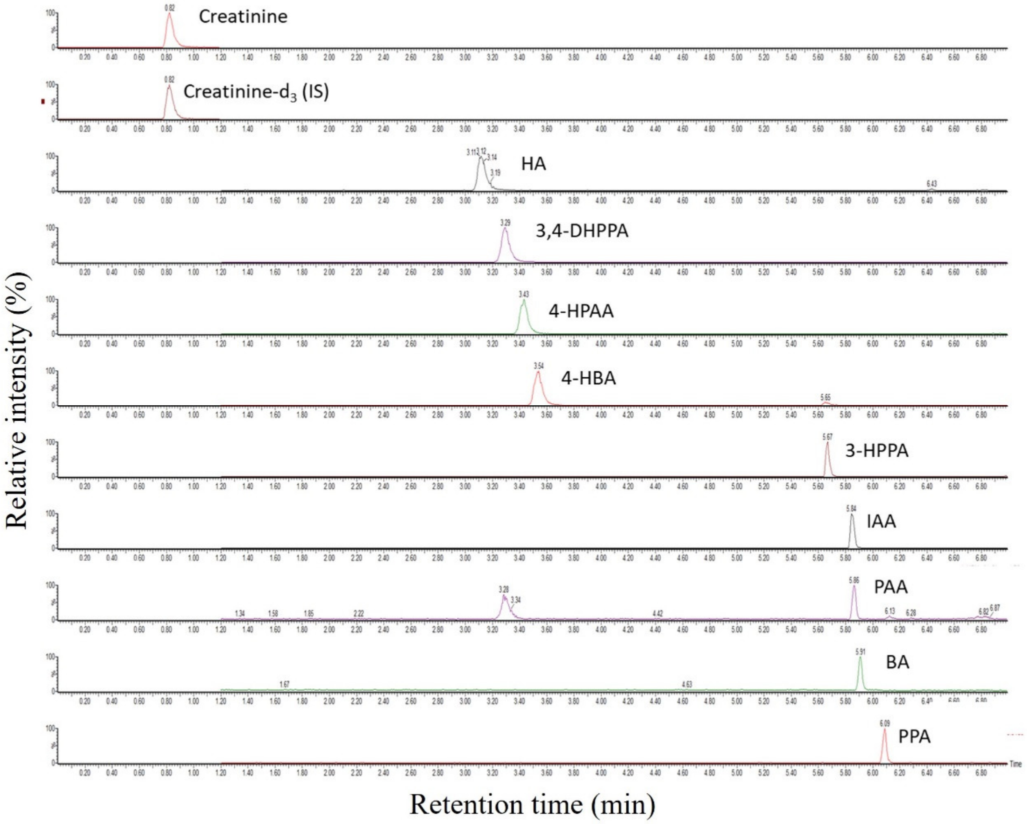

Figure S2. Representative MRM chromatograms of the nine organic acids, creatinine and creatinine-d3 (IS) standards.

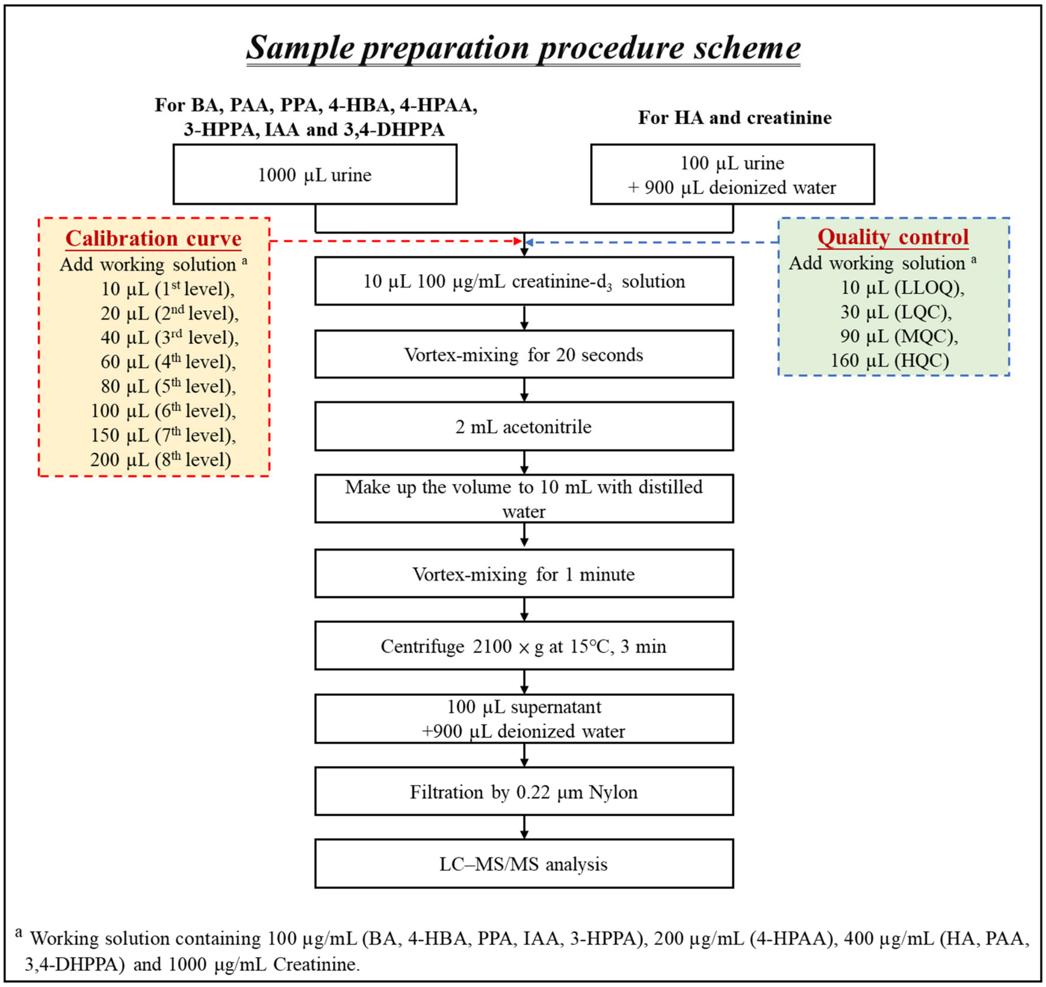

Figure S3. Sample preparation procedure scheme by gut microbiota dysbiosis related organic acids in urine.

Table S1. The detailed preparation method of calibration curve

Calibration curve preparation		1st level	2nd level	3rd level	4th level	5th level	6th level	7th level	8th level
Working solution [a] (μL)		10	20	40	60	80	100	150	200
100 μg/mL Creatinine-d_3 (IS) (μL)		10	10	10	10	10	10	10	10
Urine (mL)	For HA and Creatinine	0.1	0.1	0.1	0.1	0.1	0.1	0.1	0.1
	For others	1	1	1	1	1	1	1	1
Acetonitrile (mL)		2	2	2	2	2	2	2	2
dd H_2O quantify (mL)		10	10	10	10	10	10	10	10
The urinary sample was shaken for 1 min and centrifuged (2100 × g at 15°C, 3 min). Then, 100 μL of supernatant was diluted with 900 μL of deionized water as urinary extract. After filtration (nylon 0.22 μm), an aliquot of 5 μL filtrate was injected into the LC–MS/MS system.									

[a] Working solution containing 100 μg/mL (BA, 4-HBA, PPA, IAA, 3-HPPA), 200 μg/mL (4-HPAA), 400 μg/mL (HA, PAA, 3,4-DHPPA) and 1000 μg/mL Creatinine.

The nominal concentrations of level 1 to 8 were 10, 20, 40, 60, 80, 100, 150, and 200 ng/mL for BA, PPA, 4-HBA, 3-HPPA and IAA; 20, 40, 80, 120, 160, 200, 300, and 400 ng/mL for 4-HPAA; 40, 80, 160, 240, 320, 400, 600, and 800 ng/mL for HA, PAA, and 3,4-DHPPA; 100, 200, 400, 600, 800, 1000, 1500, and 2000 ng/mL for creatinine.

Reference

1. Bouatra, S.; Aziat, F.; Mandal, R.; Guo, A. C.; Wilson, M. R.; Knox, C.; Bjorndahl, T. C.; Krishnamurthy, R.; Saleem, F.; Liu, P. The human urine metabolome. *PLoS One* **2013**, *8*, e73076. https://doi.org/10.1371/journal.pone.0073076.
2. Jones, M. G.; Cooper, E.; Amjad, S.; Goodwin, C. S.; Barron, J. L.; Chalmers, R. A. Urinary and plasma organic acids and amino acids in chronic fatigue syndrome. *Clin. Chim. Acta* **2005**, *361*, 150-158. https://doi.org/10.1016/j.cccn.2005.05.023.
3. Loke, W. M.; Jenner, A. M.; Proudfoot, J. M.; McKinley, A. J.; Hodgson, J. M.; Halliwell, B.; Croft, K. D. A metabolite profiling approach to identify biomarkers of flavonoid intake in humans. *The Journal of nutrition* **2009**, *139*, 2309-2314. https://doi.org/10.3945/jn.109.113613.
